# Respiratory Health and Suspected Asthma among Hired Latinx Child Farmworkers in Rural North Carolina

**DOI:** 10.3390/ijerph17217939

**Published:** 2020-10-29

**Authors:** Gregory D. Kearney, Thomas A. Arcury, Sara A. Quandt, Jennifer W. Talton, Taylor J. Arnold, Joanne C. Sandberg, Melinda F. Wiggins, Stephanie S. Daniel

**Affiliations:** 1 Brody School of Medicine, East Carolina University, Greenville, NC 27834, USA; 2 Wake Forest School of Medicine, Wake Forest University, Winston-Salem, NC 27157, USA; tarcury@wakehealth.edu (T.A.A.); squandt@wakehealth.edu (S.A.Q.); jtalton@wakehealth.edu (J.W.T.); tjarnold@wakehealth.edu (T.J.A.); jsandber@wakehealth.edu (J.C.S.); sdaniel@wakehealth.edu (S.S.D.); 3 Student Action with Farmworkers, Durham, NC 27705, USA; mwiggins@duke.edu

**Keywords:** children, Hispanic, occupational, health disparities, spirometry, nitric oxide

## Abstract

The aim of this study was to evaluate respiratory health problems, including suspected asthma, and healthcare provider utilization among a sample of Latinx hired child farmworkers in rural North Carolina (n = 140). In 2018, a respiratory health questionnaire and breathing tests were collected from Latinx child (11–19 years) farmworkers (35.0% girls and 65.0% boys). Overall, 21.4% of children reported having been told by a medical provider that they had asthma, yet based on a combination of responses to respiratory survey questions, 36.4% or 15% more were identified as having suspected asthma. While 56.4% reported having a regular medical doctor, 38% had not had a medical exam in the past year. Respiratory dysfunction, including suspected, or uncontrolled asthma was prevalent among this group. Latinx children working in agriculture are vulnerable to occupational hazards and exposures and require assurances that they will receive access to high quality healthcare services that include routine respiratory health screenings.

## 1. Introduction

For child workers, agriculture is the most dangerous industry in the United States (U.S.), accounting for 42% of all youth work-related deaths [1,2]. Despite hazardous working conditions, children can work in agriculture at a far younger age than children working in non-agricultural jobs [3]. Current federal law allows children 10 or 11 years of age to work on farms not operated by their relatives, if they are engaged in non-hazardous jobs outside of school hours with parental permission [4]. Children as young as 12 or 13 can work any non-hazardous farm job outside school hours with either parental permission or on the same farm where a parent is working. Children 14 or 15 years of age can hold any non-hazardous farm job outside school hours and youths 16 and older can work any farm job, hazardous or non-hazardous, for any length of time [2,4].

It is not known how many hired children between the ages of 10 to 17 years of age work on U.S. farms [5]. The best estimates from federal government sources report that upwards of 80,000 youth, most of whom are Latinx, perform farmwork annually [6,7]. In North Carolina (N.C.), Latinx children represent the majority of hired child farmworker labor [8]. Like their agricultural working parents and caregivers, Latinx children are often hired to perform similar agricultural jobs, thereby increasing their risk and exposure to many common occupational respiratory hazards including dust, mold, pesticides, disinfectants, fertilizers, and toxic gases [9,10].

Few studies have evaluated respiratory health and asthma among rural children working in agriculture. However, in adult agricultural worker studies, respiratory illnesses and diseases have been found to be among the most common clinically diagnosed problems [10,11], and have been linked to health issues including excessive cough, congestion, wheezing, asthma-like symptoms, chronic obstructive airways disease, hypersensitivity pneumonitis, organic dust toxic syndrome (ODTS), and even death [9,12,13,14].

Latinx children hired to work on farms are a highly vulnerable group that are often confronted with potential occupational exposures that threaten their health, sometimes requiring immediate medical attention and treatment [5]. Previous studies have described disproportional health care needs among migratory agricultural farmworker families, citing barriers to access, lack of transportation and knowledge of where to go for care [15,16,17]. Given their vulnerability and life challenges, a paucity of reliable information and few published studies have ever investigated respiratory health problems as an issue among rural Latinx children. The primary purpose of this analysis is to document respiratory health problems and frequency of healthcare service visits among a sample of Latinx children in N.C. hired to perform farmwork.

## 2. Materials and Methods

### 2.1. Study Overview

This analysis uses data from the clinical component of the *Hired Child Farmworker Study*, a longitudinal, community-based participatory research study investigating the effects of farm work on the health and development of Latinx child farmworkers in N.C. [8,18]. The study is a collaboration of investigators at Wake Forest School of Medicine, Student Action with Farmworkers, and East Carolina University. This study received protocol study approvals from Wake Forest School of Medicine and East Carolina University Institutional Review Boards prior to any data collection. A more detailed discussion of the overall study development and design is presented elsewhere by Arcury and colleagues [8].

### 2.2. Participants

Participants were recruited with the help of N.C. community partner organizations that provide services to farmworkers. At the initial baseline (May to November 2017), a total of N = 202 participants were enrolled in the study. The inclusion criteria for study participants were (1) aged 10 to 17 years at recruitment, (2) self-identified as Latinx, (3) employed to do farm work in the past three months, and (4) fluent in Spanish or English. The study had no exclusion criteria. Signed parental permission and youth assent for each participant was obtained at baseline enrollment. At baseline, all 202 participants were farmworkers. In 2018, some children in the cohort had transitioned out of farm work, resulting in a sample that included both current and former rural child farmworkers.

Between July and September 2018, a subset 156 of the baseline child farmworker participants attended a clinic evaluation at locations throughout N.C. Sixteen children either refused, were unavailable to participate, or could not properly perform spirometry, resulting in a final sample size of *n* = 140 participants completing both clinic questionnaires and breathing tests.

### 2.3. Data Collection

**Figure 1:**
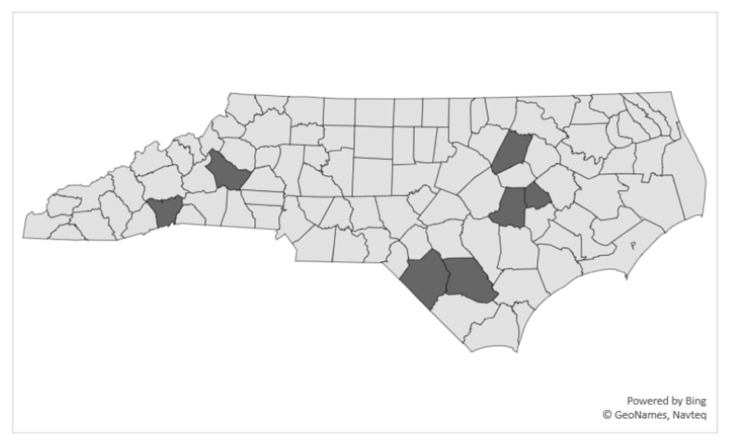
Map of North Carolina hired child Latinx farmworker study clinic sites, 2018.

The study team collected data on 10 weekends (July to September 2018) in seven N.C. counties (Figure 1). The locations of clinic sites were selected based on the number of participants in each geographical area. In areas with high numbers of participants, two clinics, several weeks apart, were scheduled to accommodate attendance. Data collection sites were locations such as community colleges, schools, and healthcare centers with multiple rooms to separate data collection activities. An interviewer-administered questionnaire was completed by participants that included items taken from other questionnaires, other validated respiratory assessments involving children [1,19,20] and those developed by the investigators [5,21]. The English version of the questionnaires was translated to Spanish, and back-translated to ensure item accuracy and reviewed by members of the Professional Advisory Committee. Pre-test interviews were conducted by study staff with members of the Youth Advisory Committee, as well as by the field interviewers with youth who had formerly worked in agriculture. Questionnaire item wording was adjusted based on feedback received during pretesting. During the clinic evaluation process, participants visited four separate data collection stations, that included, (1) a brief interview; (2) breathing tests; (3) height and weight measurements. Transportation to the clinic was provided for participants when needed. For the few participants who were unable to attend a clinic evaluation, interviewers travelled to the participants’ homes.

Breathing test data were collected by spirometry technicians and stored on password protected laptop computers. Questionnaire data were collected in booklets, verified, and then entered in Research Electronic Data Capture (REDCap) software (https://redcapinfo.ucdenver.edu/citing-redcap.html), hosted at Wake Forest School of Medicine [22,23]. REDCap is a secure, web-based survey application designed to support data capture for research studies. Participants were given an incentive of $40 for attending the clinic component of the study.

### 2.4. Materials

For anthropometric tests, participants were first asked to remove their shoes, hats, and any objects from their pockets for the measurements. Height measurements were taken in centimeters (cm) using a portable stadiometer (SECA 213, 2018, Hamburg, Germany). Interviewers recorded at least two separate height measurements. If the two measurements were more than 0.5 cm apart, then a third measurement was taken and averaged. For each participant, a single weight measurement was taken in kilograms (kg) using a digital, medical-grade portable weigh scale (TANITA BWB-800, 2011, Tokyo, Japan).

Next, participants completed a questionnaire, and performed fractional exhaled nitric oxide (FeNO) and spirometry breathing tests independently and in accordance with nationally accepted guidelines [24,25]. FeNO tests were conducted using the NIOX VERO^®®^ device (Circassia Pharmaceuticals, Inc., Morrisville, NC, USA), a rapid, non-invasive biomarker test for estimating eosinophilic airway inflammation, a factor in the causal pathway of asthma [25]. FeNO tests were performed prior to lung function tests, as spirometry maneuvers have been shown to transiently reduce exhaled nitric oxide levels [25]. Per instructional guidelines, each subject was asked to inhale to full lung capacity through the device and then exhale using a slow controlled breath for 10 s at an expiratory flow of 50 mL/s. Visual and auditory signals assisted the participant with maintaining proper flow rate. Because the device is nitric sensitive, participants were asked if they had eaten, drunk, or smoked within the past hour prior to the test. Those who answered “yes” were asked to wait one hour before testing.

Spirometry tests were performed using the Koko (nSpire Health, Longmont, CO, USA) handheld system connected to a laptop computer. Calibration of spirometry instrumentation was conducted using a Hans Rudolph, Inc., 3-L syringe (Shawnee, KS, USA). Each participant was seated, wore nose clips, and was properly coached during the procedure to forcefully exhale on three separate maneuvers. Spirometry technicians explained the purpose of the test, testing procedures, conducted all respiratory testing and reviewed the results with each participant. In situations where spirometry test results were not “normal” and/or FeNO values exceeded the recommended American Thoracic Society (ATS) cut-point level, an investigative team member informed the participant, followed by a mailed informational letter (in Spanish and English) to the child’s parent or caregiver. The letter explained the results of the tests and provided a recommendation for the parent/caregiver to take the child and a copy of the test results to a local healthcare provider for further evaluation. A list of low-cost healthcare clinics and providers located near the child’s residence was included.

### 2.5. Measures

Measures included participant’s personal and work characteristics, living arrangements, lifestyle, general health services, respiratory characteristics and breathing test results. Personal characteristics included gender (girl/boy), and age (11–15 years, and 16–19 years). Anthropometric measures included height (cm), weight (kg), and body mass index z-score (BMI-Z). Dichotomous measures (yes/no) were used for “migrant worker,” which is whether the participant changed residence for agricultural employment, and “unaccompanied,” in which the participant lived with neither their father nor mother.

Work characteristics included dichotomous measures (yes/no) for crops which the participant worked in the past week (i.e., tobacco, berries, tomatoes, sweet potatoes, green peppers, squash, hot peppers, cucumbers, melons, and other) and were only posed to those participants who reported doing farm work in the “current year” (2018). Participant’s general health characteristics included yes/no responses to whether the child had a regular medical doctor and had received a medical exam in past year.

### 2.6. Respiratory Health and Suspected Asthma Questionnaire

Measures to assess breathing problems and suspected asthma were evaluated using a nine-item screening questionnaire that recorded each participant’s reported response to yes/no questions (e.g., “Has a doctor ever told you that you had asthma?”) The number of yes/no responses were totaled, and participants were classified as either having, “no evidence of asthma,” “previously diagnosed or current asthma,” or “suspected, undiagnosed or uncontrolled asthma.”

### 2.7. Spirometry and FeNO

Spirometric lung function measures included forced vital capacity (FVC), forced expiratory volume in the first second (FEV_1_), and FEV_1_/FVC ratio. The results were expressed as the percentage of predicted normal values, and were categorized as either, “normal spirometry values,” “possible early obstructive impairment,” or “restrictive.” In spirometry, “obstructive” and “restrictive” results are universally recognized as abnormality classification patterns to signify airway impairment. “Obstructive” lung function is a hallmark indicator of asthma, while “restrictive” is more commonly associated with non-asthma respiratory diseases (i.e., pulmonary fibrosis and pneumoconiosis) [14]. Lung function test values were compared to national reference level values (Hankinson) for Mexican Americans [26]. Interpretations of spirometry tests were made based on the ATS clinical standard, 5th percentile of the predicted value, or lower limit of normal (LLN) [27].

Measurements for airway inflammation (FeNO) were established using the ATS recommended guidelines (2011) in persons 12 years of age or older [28]. Cut-points measurements rather than reference values were used when interpreting FeNO levels and were defined as low (<25 ppb), intermediate (25–50 ppb), and high (50 ppb) [25,28].

### 2.8. Analysis

Descriptive statistics (count, percent) were calculated for personal, work characteristics and reported respiratory health symptoms. Means and standard deviations were calculated for anthropometric, respiratory characteristics, and breathing tests within the sample. Associations between these measures and gender were calculated using t-tests. The associations between gender and spirometry and FeNO measures were examined using Chi-square or Fisher’s Exact tests as appropriate. All analyses were performed using SAS v 9.4 (SAS Institute, Cary, NC, USA), and *p*-values of < 0.05 were considered statistically significant.

## 3. Results

### 3.1. Personal and Work Characteristics

Forty-nine or 35.0% of participants were girls, and 91 or 65.0% were boys (Table 1). In age categories, 45% of children were 11–15 years old, and 55% were 16–19 years of age. Fifty participants (35.7%) reported working with an adult relative or parent, 5.0% were unaccompanied (living with neither their mother nor father), and 13.6% reported being a migrant.

Children worked across a variety of crops during the year of the study, including tobacco (29.3%), berries (19.3%), tomatoes (13.6%), sweet potatoes (9.3%), green peppers (3.6%), squash (2.9%), hot peppers (4.3%), cucumbers (3.6%), melons (2.9%), and other crops (6.4%). Only three (2.1%) participants either smoked, chewed or used snuff tobacco “sometimes” or “often.” Seventy-nine or 56.4% had seen a regular medical doctor, and 87 (62.1%) had had a medical exam in the past year.

### 3.2. Respiratory Health Characteristics

When reporting respiratory health symptoms, 14.3% of participants had ever had a cough that lasted more than 10 days, and 16.4% reported ever experiencing wheezing or whistling in the chest (Table 2.) Twenty-seven, or 19.3% had had to stop running or playing because of coughing or wheezing, 30.7% indicated their chest had ever felt tight, heavy or hurt, almost 13.6% had breathing problems that woke them up at night, and 6.4% had experienced breathing problems when they first woke up in the morning. Thirty or 21.4% of participants reported ever being told by a doctor that they had asthma, and 8.6% currently took asthma medicine prescribed by a doctor; 4.3% had been told by a healthcare provider that they had bronchitis.

Based on the summation of yes/no responses to respiratory questions, 43.6% of children reported having zero respiratory problems while 24.3% reported one respiratory problem and 45 or 32.1% reported two or more respiratory problems. When combining selected questions to determine asthma status, 78 or 55.7% had “no evidence of asthma,” 11 or 7.9% had “previous or current asthma,” and 51 or 36.4% had “suspected asthma.”

### 3.3. Age, Anthropometric and Lung Function Values

Girls and boys had similar ages, with a mean age among girls of 15.76 years and 15.64 years for boys (Table 3). BMI z-scores were slightly lower for girls (0.95) than for boys (0.99), but not significantly different. Average height was significantly less for girls (156.18 cm) than for boys (167.10 cm), and average weight was more for boys (72.75 kg) than for girls (64.46 kg).

In spirometry, overall, mean lung function values (observed, predicted, percentage predicted) for FVC and FEV_1_ were within predicted value ranges and differences between genders were statistically significant (all *p* ≤ 0.01), with boys having higher lung function values compared to girls. The observed and predicted mean FEV_1_/FVC ratio values for girls were significantly higher than for boys, but the significant difference did not hold for the percentage predicted values; lung function values were within 5% of the predicted ratio.

### 3.4. Spirometry and FeNO

Overall, 77.1% of all children in our sample had reports of “normal” spirometry. This included 79.6% of all girls and 75.8% of all boys (Table 4). Thirty, or 21.4%, had reports of obstructive or suspected asthma, with a higher percentage among boys than girls (23.1% vs. 18.4%). Two or 1.4% of participants had restrictive test results.

As shown in Table 5, 66.2% of participants’ FeNO values were less than 25 ppb, nearly 20% of children had values between 25 and 50 ppb, and 19 (14.0%) of children had FeNO levels higher than 50 ppb. There was not a significant difference in FeNO values between genders (*p* = 0.22).

## 4. Discussion

This evaluation among a sub-sample of participants identified that nearly one-third of hired Latinx child farmworkers had two or more breathing problems, and over 36% had suspected asthma. The use of thorough assessment tools, including more in-depth, structured questions accompanied by spirometry detected a significant high prevalence of breathing problems, including possible undiagnosed or uncontrolled asthma among these children.

In the present study, wheezing was among the most common respiratory problem with nearly 20% of children reporting it. In one of the few comparative studies available, Garcia and colleagues found similar results among adolescent Hispanic farmworkers working in low-lying crops in Indiana with a high percentage of cough symptoms (22.5%) but lower for wheezing (11.1%) [29]. In a separate adult agricultural worker study, Mirabelli and colleagues also found a high percentage of wheezing among Latinx farmworkers that had been working in tobacco within the past month [30]. It is certainly plausible that these children experienced increased respiratory symptoms as a result of exposure to activities related to farmwork. However, because these children performed multiple job tasks, including working in tobacco and other crops, during various time periods it was difficult to assess respiratory symptoms with specific work exposure activities. Nevertheless, given our findings of the high percentage of documented respiratory problems among these children, further investigation of this relationship and causal exposure pathways need to be explored.

### 4.1. Factors Related to Asthma

The number that self-reported as previously diagnosed or with current asthma was 7.9%. In 2018, the Centers for Disease Control and Prevention (CDC) estimated lifetime asthma prevalence among Mexican/Mexican American children (less than 18 years) at 11.5%, or 12.0% among boys and 11.0% for girls [31]. Although overall asthma prevalence was found lower in this study compared to the national average, spirometry results among this sample highly differed, with over 18% of girls and 23% of boys identified with airflow obstruction or having suspected asthma.

Participants reporting currently taking asthma medicine was 8.6%. Whether or not those participants reporting having asthma were taking their medication as prescribed could not be determined. As expected, FeNO test levels varied across all participants. Overall, 30% of participants exceeded the recommended 25-ppb FeNO cut point indicator for eosinophilic activity, or airway inflammation. In some cases, participants including both those with reported asthma and suspected asthma reached FeNO levels of over 100 ppb. While these high levels are very concerning, they should be interpreted with caution. FeNO represents a good biomarker for detecting airway inflammation, but high FeNO levels alone are not a sole indicator for diagnosing asthma [20,25,28]. Other factors including allergies or a having a cold can also increase eosinophil activity and influence FeNO levels.

Without more information it is difficult to determine what specific factors are contributing to these children’s poor respiratory health. Estimating statistical associations between crop type worked and respiratory problems were evaluated by the investigators but proved somewhat problematic given that children worked in a variety of crops and at different lengths of time. The combination of genetics and environmental factors are valid explanations for these children’s breathing problems and asthma [20], but disparities in healthcare, such as lack of insurance coverage, quality of care, utilization (i.e., including routine well-care visits), and asthma management are more likely reasons. Rosser and colleagues found that Latinx tend to be less likely to use asthma controller medicine than whites, largely attributed to parental beliefs concerning side effects, medication costs, language barrier, and low expectations for asthma control [32]. Without proper asthma management, guidance, and trust by caregivers, children and their parents have been shown to be less likely to comply with asthma medication [33].

Contrary to earlier studies that report healthcare access and utilization as barriers to farmworkers and their children [16,17], the parent study of this project found a surprisingly high level of health service utilization among children [5]. Recruitment of study participants by community-partner health and rural outreach clinics may help to explain these higher rates. However, given that the current study identified a high percentage of suspected asthma and breathing problems, leads us to believe that many of these children had never had a proper medical respiratory evaluation. A little more than half of the children (56.4%) surveyed had a regular medical doctor, yet almost 40% of children had not had a healthcare visit within the past year. This suggests to us that many of these children may not have ever received a necessary recommended well-childcare visit. Well-care visits include comprehensive assessments of health and health-related issues, including breathing tests. While some of these children may have experienced an impromptu healthcare provider visit, for example for an acute injury, some of them may have never been diagnostically assessed for asthma.

### 4.2. Strengths and Limitations

This study uses a combination of a respiratory questionnaire, spirometry, and FeNO in a non-clinical setting to evaluate breathing problems among hired Latinx child farmworkers. The asthma screening questionnaire was simple and convenient to use for quickly assessing breathing problems, while breathing tests provided an added diagnostic measurement for assessing lung health. While these tools proved useful, a more thorough assessment for asthma should include a bronchodilator challenge test, something which we did not include.

Because the study focused on Latinx child farmworkers in only one state and with a relatively small sample size, it limits the generalizability of the findings. Participants self-reported which could imply bias and limits the accuracy of these results.

## 5. Conclusions

Major findings of this study identified that many of these rural children hired to do farmwork experience respiratory dysfunction and have undiagnosed, or uncontrolled asthma. Many of them do not receive recommended annual well-childcare visits which likely explains the higher respiratory problems. More in-depth research is needed to better evaluate these children’s utilization of healthcare services, cultural beliefs and respiratory problems associated with occupational exposures and specific job activities, such as working in tobacco.

Rural, hired Latinx child farmworkers are vulnerable to many life and work obstacles [34]. Being hired to perform potentially hazardous and dangerous farm jobs is work that should not be performed by children. Because of their young age, children are sensitive to harmful exposures, accidents and injuries that can result in unfortunate life-long health consequences [35].

Preventive strategies to address child labor and exploitation among hired youth farmworkers should include policy changes to increase the age limit and decrease the number of work hours in farm labor. Providing higher pay to adult farmworker families could help narrow the current wage gap and reduce the number of hired children to work on farms. Until fewer children are hired to do farmwork, increased efforts to closely monitor the safety and respiratory health of this high-risk group must be a priority.

## Figures and Tables

**Table 1:** Participant personal, work and health characteristics, hired Latinx child farmworkers in North Carolina, 2018.

Personal and Work Characteristics	n = 140 *n* (%)
Gender	
Girls	49 (35.0)
Boys	91 (65.0)
Age (years)	
11	3 (2.1)
12	4 (2.9)
13	13 (9.3)
14	19 (13.6)
15	24 (17.1)
16	20 (14.3)
17	29 (20.7)
18	26 (18.6)
19	2 (1.4)
Age Groups (years)	
11–15	63 (45.0)
16–19	77 (55.0)
Work/Living Arrangement	
Works in farmwork with an adult relative (including parent) ^1^	50 (35.7)
Unaccompanied (lives with neither father nor mother)	7 (5.0)
Migrant worker (moves from state-to-state to do farmwork) ^1^	19 (13.6)
Crops worked in past week ^1,2^	
Tobacco	41 (29.3)
Berries	27 (19.3)
Tomatoes	19 (13.6)
Sweet potatoes	13 (9.3)
Green peppers	5 (3.6)
Squash	4 (2.9)
Hot peppers	6 (4.3)
Cucumbers	5 (3.6)
Melons	4 (2.9)
Other	9 (6.4)
Lifestyle ^3^	
Smoke, chew, or use snuff tobacco	3 (2.1)
Health Services	
Has regular medical doctor	79 (56.4)
Had medical exam in past year	87 (62.1)

Notes: ^1^ Fifty-eight children were not working in farmwork during the summer this questionnaire was administered. Thus, they were considered as a “no” response for these questions. ^2^ Other crops include peas, cotton, green beans, okra, hay, herbs, soybeans. Some children worked in multiple crops. ^3^ Smoke, chew or snuff tobacco includes participants that responded either “sometimes” or “often”.

**Table 2:** Respiratory health questions, number of respiratory health problems and asthma status classification, hired Latinx child farmworkers in North Carolina, 2018.

Respiratory Health Parameters	n = 140 *n* (%)
1. Ever had a cough that would not go away or lasted more than 10 days?	20 (14.3)
2. Ever experienced wheezing or whistling in the chest?	23 (16.4)
3. Ever had to stop running or playing because of coughing or wheezing?	27 (19.3)
4. Has your chest ever felt tight, heavy or hurt?	43 (30.7)
5. Have you ever had breathing problems (coughing, wheezing, shortness of breath, chest tightness) that woke you up at night	19 (13.6)
6. Have you ever had breathing problems (coughing, wheezing, shortness of breath, chest tightness) when you first woke up in the morning?	9 (6.4)
7. Has a doctor ever told you that you have asthma?	30 (21.4)
8. Do you take asthma medicine prescribed by a doctor daily or even occasionally?	12 (8.6)
9. Has a doctor or medical professional ever said you had bronchitis?	6 (4.3)
Number of Respiratory Health Problems Reported	
0	61 (43.6)
1	34 (24.3)
2 or more	45 (32.1)
Asthma status classification	
No evidence of asthma	78 (55.7)
Previously diagnosed or current asthma	11 (7.9)
Suspected undiagnosed asthma	51 (36.4)

Notes: Asthma status was classified as follows; No evidence of asthma: all “no” answers, “yes” to question 2 only, “yes” to question 4 only, or “yes” to questions 2 and 9 only; Previous or current asthma: answer “yes” to questions 7 and 8; Suspected, undiagnosed asthma: all other combinations of answers.

**Table 3:** Mean values of age, anthropometric and lung function values, hired Latinx child farmworkers in North Carolina (n = 140).

Parameters	Total		Girls (n = 49)		Boys (n = 91)		
	*M*	*SD*	*M*	*SD*	*M*	*SD*	*p*
Anthropometric							
Age	15.68	1.88	15.76	1.97	15.64	1.84	0.73
BMI (z-scores)	0.97	1.13	0.95	1.04	0.99	1.19	0.84
Height (cm)	163.28	8.7	156.18	6.94	167.10	7.00	<0.0001
Weight (kg)	69.85	19.05	64.46	18.53	72.75	18.78	0.01
Lung Function Values (L)							
FVC							
Observed	4.22	0.95	3.37	0.54	4.68	0.79	<0.0001
Predicted	4.04	0.65	3.41	0.35	4.38	0.50	<0.0001
Percentage Predicted	1.05	0.17	1.00	0.11	1.07	0.18	<0.01
FEV_1_							
Observed	3.60	0.74	2.96	0.41	3.94	0.64	<0.0001
Predicted	3.53	0.53	3.03	0.30	3.81	0.42	<0.0001
Percentage Predicted	1.02	0.15	0.99	0.11	1.04	0.16	0.01
FEV_1_/FVC							
Observed	0.86	0.07	0.88	0.07	0.85	0.07	<0.01
Predicted	0.87	0.01	0.89	0.01	0.87	0.01	<0.0001
Percentage Predicted	0.98	0.08	1.00	0.08	0.98	0.08	0.22

Notes: Liters (L); FVC (Forced Vital Capacity); FEV_1_ (Force Expiratory Volume in first second); FEV_1_/FVC ratio.

**Table 4:** Spirometry, hired Latinx child farmworkers in North Carolina (n = 140).

Spirometry classification ^1^	Total		Girls n = 49		Boys n = 91		
	*n*	%	*n*	%	*n*	%	*p*
Normal	108	77.1	39	79.6	69	75.8	0.71
Obstructive (suspected asthma)	30	21.4	9	18.4	21	23.1	
Restrictive	2	1.4	1	2.0	1	1.1	

Notes: ^1^ Normal, Obstructive and Restrictive classifications were derived using interpretations generated by computer spirometry reports.

**Table 5:** FeNO, hired Latinx child farmworkers in North Carolina (n = 136).

Parameters	Total		Girls n = 48		Boys n = 88		
	*n*	%	*n*	%	*n*	%	*p*
FeNO (ppb)n ^1^							
<25	90	66.2	36	75.0	54	61.4	0.22
25–50	27	19.8	6	12.5	21	23.9	
>50	19	14.0	6	12.5	13	14.8	

Notes^: 1^ High FeNO includes participants with results > 25 ppb. FeNO tests did not include three participants < 12 years old.
